# Estimation of Melanin and Hemoglobin Using Spectral Reflectance Images Reconstructed from a Digital RGB Image by the Wiener Estimation Method

**DOI:** 10.3390/s130607902

**Published:** 2013-06-19

**Authors:** Izumi Nishidate, Takaaki Maeda, Kyuichi Niizeki, Yoshihisa Aizu

**Affiliations:** 1 Graduate School of Bio-applications and Systems Engineering, Tokyo University of Agriculture and Technology, 2-24-16, Naka-cho, Koganei-shi, 184-8588 Tokyo,Japan; 2 Department of Mechanical Engineering, Kushiro National College of Technology, 2-32-1, Otanoshike-Nishi, Kushiro-Shi, 084-0916 Hokkaido, Japan; E-Mail: t-maeda@mech.kushiro-ct.ac.jp; 3 Graduate School of Bio-system Engineering, Yamagata University, 4-3-16, Jonan, Yonezawa-Shi, 992-8510 Yamagata, Japan; E-Mail: nzq@yz.yamagata-u.ac.jp; 4 College of Design and Manufacturing Technology, Muroran Institute of Technology,27-1 Mizumoto-cho, Muroran-shi, 050-8585 Hokkaido, Japan; E-Mail: aizu@mmm.muroran-it.ac.jp

**Keywords:** multi-spectral imaging, diffuse reflectance, skin, melanin, oxygenated hemoglobin, deoxygenated hemoglobin, blood, tissue oxygen saturation, multiple regression analysis, Monte Carlo simulation

## Abstract

A multi-spectral diffuse reflectance imaging method based on a single snap shot of Red-Green-Blue images acquired with the exposure time of 65 ms (15 fps) was investigated for estimating melanin concentration, blood concentration, and oxygen saturation in human skin tissue. The technique utilizes the Wiener estimation method to deduce spectral reflectance images instantaneously from an RGB image. Using the resultant absorbance spectrum as a response variable and the extinction coefficients of melanin, oxygenated hemoglobin and deoxygenated hemoglobin as predictor variables, multiple regression analysis provides regression coefficients. Concentrations of melanin and total blood are then determined from the regression coefficients using conversion vectors that are numerically deduced in advance by the Monte Carlo simulations for light transport in skin. Oxygen saturation is obtained directly from the regression coefficients. Experiments with a tissue-like agar gel phantom validated the method. *In vivo* experiments on fingers during upper limb occlusion demonstrated the ability of the method to evaluate physiological reactions of human skin.

## 1. Introduction

Quantitative evaluation of the melanin and blood concentrations and the blood oxygenation is important for detecting various skin diseases including cancers, monitoring health status and tissue metabolism, and evaluating convalescence. Major chromophores in the superficial skin layer are melanin, oxygenated hemoglobin, and deoxygenated hemoglobin, which show distinctive optical absorption properties in the visible wavelength range. If the concentration of each chromophore varies, then, the corresponding change may be made on diffusely reflected light from the skin tissue in this wavelength range. Therefore, analysis of the diffuse reflectance spectra may provide us with useful information on tissue activities and functions that are related to the melanin and hemoglobin. Diffuse reflectance spectroscopy (DRS) has been widely used for the evaluation of human skin chromophores at a single location [1–10]. Multi-spectral imaging based on diffuse reflectance spectroscopy has been widely employed for evaluating the spatial distribution of chromophore contents in living tissue using a series of discrete narrow-band filters or a liquid crystal tunable filter [11–13]. A simple method for imaging concentrations of melanin and blood and imaging oxygen saturation in human skin tissue based on the diffuse reflectance images at six wavelengths (500, 520, 540, 560, 580 and 600 nm) using multiple regression analysis aided by the Monte Carlo simulations has been previously proposed [14]. In this method, however, use of a monochromatic charge coupled device (CCD) camera with mechanical rotatory filters required 12 s for reflectance images at the six wavelengths.

To achieve rapid multispectral imaging, use of an acousto-optical tunable filter [15] and the combination of a lenslets array with narrow-band filters [16] have been proposed. On the other hand, the reconstruction of multispectral images from a red green blue (RGB) image acquired by a digital RGB camera is promising to perform rapid and cost-effective multispectral imaging. Several reconstruction techniques for the multispectral images, such as pseudo-inverse method [17–20], finite-dimensional modeling [19,21], nonlinear estimation method [22], and Wiener estimation method (WEM) [23–25] has been studied. Among these reconstruction techniques, WEM is one of the most promising methods for practical uses because of its simplicity, cost-effectiveness, accuracy, time efficiency, and possibility of high resolution image acquisition. In this paper, simple and cost-effective multi-spectral imaging based on that method [14] designed to estimate melanin concentration, blood concentration, and oxygen saturation in the skin using the spectral reflectance images reconstructed from a single snap shot of RGB image by WEM is described.

## 2. Principle

### 2.1 Reconstruction of Spectral Image by Wiener Estimation Method

The response of a digital color camera in spatial coordinates (*x*, *y*) with *i*th (*i* = 1, 2, 3) color channel, or Red, Green, and Blue, can be calculated as:

(1) $$v_{i}(x,y)=\int u_{i}(\lambda )E(\lambda )S(\lambda )r(x,y;\lambda )d\lambda$$

where *λ* is wavelength, *u_i_*(*λ*) is transmittance spectrum of the *i*th filter, *E*(*λ*) is the spectrum of the illuminant, *S*(*λ*) is the sensitivity of the camera, and *r* (*x*, *y*; *λ*) is the reflectance spectrum in the spatial coordinates (*x*, *y*). For convenience, Equation (1) is expressed by discrete vector notation as:

(2) v=Fr

where **v** is a vector with three element column and **r** is a vector with a *k* element column which corresponds to the reflectance spectrum of a pixel of an image. **F** is a 3 × *k* matrix and expressed as:

(3) F=UES

where **U** = [u_1_, u_2_, u_3_] *^T^*. Column vector *u_i_* denotes the transmittance spectrum of the *i*th filter and [ ] *^T^* represents the transposition of a vector. **E** and **S** are *k* × *k* diagonal matrices and represent the spectrum of illuminant and the sensitivity of the camera, respectively. In this study, we use the known spectral profiles of illuminant and camera which has been published by the manufacturers. The Wiener estimation of **r** is given by:

(4) $$\tilde{\text{r}}=\text{Wv}$$

where **W** is the Wiener estimation matrix. The purpose of **W** is to minimize the minimum square error between original and estimated reflectance spectrum. In this case, the minimum square error is expressed as:

(5) $$e=\langle {\left(\text{r}-\tilde{\text{r}}\right)}^{t}\left(\text{r}-\tilde{\text{r}}\right)\rangle$$

From Equations (4) and (5), the minimum square error is rewritten as:

(6) $$e=\langle {\left(\text{r}-\tilde{\text{r}}\right)}^{t}\left(\text{r}-\tilde{\text{r}}\right)\rangle =\langle {\text{r}}^{t}\text{r}\rangle -\text{W}\langle {\text{r}}^{t}\text{v}\rangle -{\text{W}}^{t}\langle {\text{v}}^{t}\text{r}\rangle +{\text{W}}^{t}\text{W}\langle {\text{v}}^{t}\text{v}\rangle$$

The minimization of the minimum square error requires the condition that partial derivative of *e* with respect to **W** is zero as:

(7) $$\frac{\partial e}{\partial \text{W}}=-\langle {\text{r}}^{t}\text{v}\rangle +{\text{W}}^{t}\langle {\text{v}}^{t}\text{v}\rangle =0$$

From the condition of Equation (7), the matrix W is derived as:

(8) $$\text{W}=\langle {\text{rv}}^{T}\rangle \;{\langle {\text{vv}}^{T}\rangle }^{-1}=\langle {\text{rv}}^{T}\rangle \;{\text{F}}^{T}{\left(\text{F}\langle {\text{rr}}^{T}\rangle {\text{F}}^{T}\right)}^{-1}$$

where, 〈 〉 is an ensemble-averaging operator. To derive the matrix **W**, the autocorrelation matrix 〈**rr***^T^*〉 is required. In this study, we determined 〈**rr***^T^*〉on the basis of 341 different reflectance spectra obtained from human skins of five volunteers (all men, mean age 23 ± 1 years; under the various physiological conditions induced by upper arm occlusions. Discrete reflectance values ranged from 450 to 690 nm at 10 nm intervals were extracted from the raw reflectance spectrum measured by spectrometer and assigned to the 25 elements of vector **r** . A matrix **rr***^T^* with 25 × 25 elements was derived by multiplying the vector **r** and its transposition vector **r***^T^*. This procedure was applied to each reflectance sample and the 341 different matrices of **rr***^T^* were derived. The autocorrelation matrix 〈**rr**^T^〉 was calculated by averaging the corresponding elements over all samples.

To test the accuracy in spectral reconstruction, the estimated spectrum by WEM is compared with the measured spectrum by spectrometer using a goodness-of -fit coefficient (GFC) [26]. The GFC is based on the inequality of Schwartz and it is described as:

(9) $$\text{GFC}=\frac{\left|\underset{j}{\text{∑}}r_{\text{mes}}(\lambda_{j})r_{\text{est}}(\lambda_{j})\right|}{\sqrt{\left|\underset{j}{\text{∑}}{\left[r_{\text{mes}}(\lambda_{j})\right]}^{2}\right|\sqrt{\left|\underset{j}{\text{∑}}{\left[r_{\text{est}}(\lambda_{j}\right]}^{2}\right|}}}$$

where *r_mes_* (*λ_j_*) is the measured original spectral data at the wavelength *λ_j_* and *r_est_* (*λ_j_*) is the estimated spectral data at the wavelength *λ_j_* . Hernández-Andrés *et al.* [26] suggested that colorimetrically accurate *r_mes_* (*λ_j_*) requires a GFC > 0.995; a “good” spectral fit requires a GFC ≥ 0.999, and GFC ≥ 0.999 is necessary for an “excellent” spectral fit.

### 2.2 Estimation of Chromophores Based on Multiple Regression Analysis

An absorbance spectrum *A*(*λ*) is defined as:

(10) $$A\left(\lambda \right)=-{\text{log}}_{10}r(\lambda )$$

where *r*(*λ*) is the diffuse reflectance spectrum normalized by the incident light spectrum. According to the Lambert-Beer law, *A* (*λ*) is expressed by the sum of absorbances due to major chromophores in skin tissue as:

(11) $$A\left(\lambda \right)=C_{m}l_{e}\left(\lambda ,C_{m}\right)\varepsilon_{m}\left(\lambda \right)+C_{\text{ob}}l_{d}\left(\lambda ,C_{\text{ob}},C_{\text{db}}\right)\varepsilon_{\text{ob}}\left(\lambda \right)+C_{\text{db}}l_{d}\left(\lambda ,C_{\text{ob}},C_{\text{db}}\right)\varepsilon_{\text{db}}\left(\lambda \right)+D\left(\lambda \right)$$

where *C* is the concentration, *l* is the mean optical path length, *ε* (*λ*) is the extinction coefficient, and *D* (*λ*) indicates attenuation due to light scattering in the tissue. Subscripts *e*, *d*, *m*, *ob* and *db* denote epidermis, dermis, melanin, oxygenated blood and deoxygenated blood, respectively. By using the absorbance spectrum as the response variable and extinction coefficients as the predictor variables the multiple regression analysis (MRA1) can be applied to Equation (11) as:

(12) $$A\left(\lambda \right)=a_{m}\varepsilon_{m}\left(\lambda \right)+a_{\text{ob}}\varepsilon_{\text{ob}}\left(\lambda \right)+a_{\text{db}}\varepsilon_{\text{db}}\left(\lambda \right)+a_{0}$$

where *a_m_*, *a_ob_*, *a_db_*, and *a_0_* are the regression coefficients [8]. The regression coefficients, *a_m_*, *a_oh_*,*a_dh_*, and *a_0_* describe the degree of contribution of each extinction coefficient to *A* (*λ*) and are closely related to the concentrations *C_m_*, *C_ob_* and *C_db_*. The total blood concentration is defined as the sum of the concentrations of oxygenated blood and deoxygenated blood *C_ob_*+*C_db_*. Tissue oxygen saturation is determined as:

(13) $$StO_{2}\%=100\times \frac{a_{ob}}{a_{ob}+a_{db}}=100\times \frac{a_{ob}}{a_{tb}}$$

Then, multiple regression analysis (MRA2) is further used with the regression coefficients obtained from Equation (12) [8]. In this case, *C_m_* and *C_tb_* are regarded as the response variables, and the 4 regression coefficients from Equation (12) and their higher order terms are regarded as the predictor variables. Thus, their relations are written as:

(14) $$C_{m}={\text{b}}_{m}.\text{a}$$

(15) $$C_{tb}={\text{b}}_{tb}\cdot \text{a}$$

(16) $$\text{a}={\left[1,a_{m},a_{tb},a_{o},a_{m}\times a_{tb},a_{m}\times a_{o},\cdots \text{higher order terms}\cdots \right]}^{T}$$

(17) $${\text{b}}_{m}=[b_{m,0},\;b_{m,1},\;b_{m,2},\cdots ,\;b_{m,z-1}]$$

(18) $${\text{b}}_{tb}=\left[b_{tb,0},\;b_{tb,1},\;b_{tb,2},\cdots ,b_{tb,z-1}\right]$$

Coefficients *b_m,i_* and *b_tb_*_,_*_i_* (*i* = 0, 1, 2, …) are unknown and must be determined before the analysis. The number of components in vectors **a**, **b***_m_* and **b***_tb_* is assumed to be *z.* Monte Carlo simulations (MCS) [27] were adopted as the foundation to establish reliable values of *b_m,i_* and *b_tb_*_,_*_i_*. In the present MCS, the absorption coefficients *μ_a_* converted from the concentrations are provided as input to simulation, while the diffuse reflectance is produced as output. The input concentrations and the output reflectance are helpful as the data set in specifying the values of *b_m,i_* and *b_tb_*_,_*_i_* statistically. The skin tissue model consisted of an epidermis and a dermis layer. The absorption coefficient of melanin *μ_a,m_* [28] for *C_m_* = 1 to 10% at intervals of 1% were input to the epidermis. The sum of the absorption coefficient of oxygenated and deoxygenated blood *μ_a,ob_*+ *μ_a,db,_* which represents the absorption coefficient of total blood *μ_a_*_,_*_otb_* for *C_tb_*= 0.2, 0.4, 0.6, 0.8, and 1.0% were input to the dermis [8]. Oxygen saturation, *StO_2_*= 0, 20, 40, 60, 80, and 100% were determined from the relation *μ_a,ob_*/*μ_a_*_,_*_tb_*. The scattering coefficient *μ_s,_* anisotropy factor *g*, refractive index *n*, the layer thickness are given as constants in this study. Typical values for *μ_s_* and *g* for both the epidermis and dermis were used [8]. The thicknesses of the epidermis and dermis were set to be 0.06 mm and 4.94 mm, respectively. The value of *n* for each layer was assumed to be 1.4. In total, 300 diffuse reflectance spectra were simulated under various combinations of *C_m,_C_tb,_* and *StO_2_* in the range from 500 to 600 nm at intervals of 20 nm. MRA1 for each simulated spectrum based on Equation (12) generated the 300 sets of vector **a** and concentrations *C_m_* and *C_tb_*. The vectors **b***_m_* and **b***_tb_* were determined by performing MRA2. The number of *z* was 14. Once **b***_m_* and **b***_tb_* were obtained, *C_m_* and *C_tb_* were calculated from *a_m,_a_tb_* and *a_0_* derived from MRA1 without MCS, for each pixel of a multi-spectral reflectance image.

## 3. Experiments

**Figure 1.**
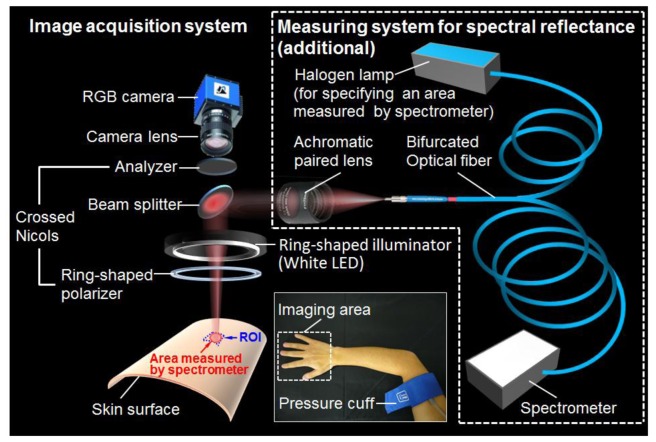
Experimental configuration for the image acquisitions and the spectral reflectance measurements.

**Figure 2.**
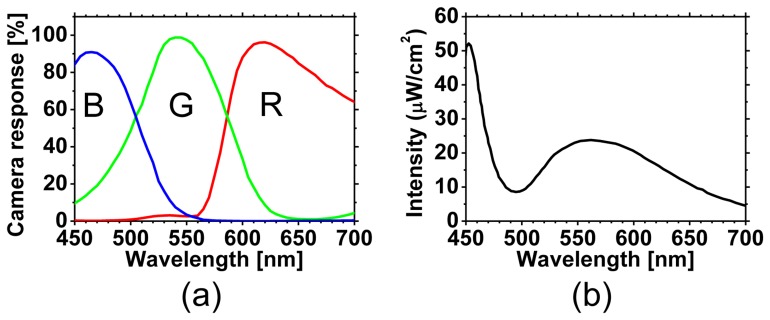
Spectral profiles for (**a**) the RGB-responses of camera and (**b**) the intensity of illuminant.

Figure 1 shows a schematic illustration of experimental system used in this study. A White light emitting diode (LED) (LA-HDF158A, Hayashi Watch Works Co., Ltd, Tokyo, Japan) illuminated the skin surface via a light guide and a ring illuminator with a polarizer. Diffusely reflected light was received by a 24-bit RGB CCD camera (DFK-31BF03, Imaging Source LLC, Charlotte, NC, USA) with a camera lens and an analyzer to acquire an RGB image of 1,024 × 768 pixels. The spectral profiles of illuminant and camera are shown in Figure 2(a,b), respectively. The primary polarization plate (ring-shaped polarizer) and the secondary polarization plate (analyzer) were set to be a crossed Nicols alignment in order to reduce specular reflection from the skin surface. A standard white diffuser was used to regulate the camera white balance. To evaluate accuracy of WEM, the reflectance spectra of skin were simultaneously measured by a fiber-coupled spectrometer (USB2000, Ocean Optics Inc., Dunedin, FL, USA) at 110 ms of integration time as reference data. Before the sequential measurements of RGB images and reflectance spectra, a measured area by the spectrometer was confirmed by projecting light from a halogen lamp on the skin surface via one lead of bifurcated fiber, lens, and beam splitter. The RGB image of skin surface including the spot of light illuminated by the halogen lamp was stored into the PC, and then, the size and coordinates of the spot were specified as the measured area by spectrometer. After the halogen lamp was turned off, the sequential measurements of RGB images and reflectance spectra were simultaneously performed. The region of interest, or ROI on the image of skin surface was selected to be the same as the measured area by the spectrometer. Using WEM, reflectance images ranged from 450 to 690 nm at intervals of 10 nm were reconstructed from a RGB image acquired at 65 ms of exposure time. This means that the spectral images at 25 wavelengths can be obtained with a temporal resolution of 15 fps. Images at 6 wavelengths (500 to 600 nm at intervals of 20 nm) were then used to estimate the *C_m_*_,_*C_tb_*_,_ and *StO*_2_ images according to the above process.

**Figure 3.**
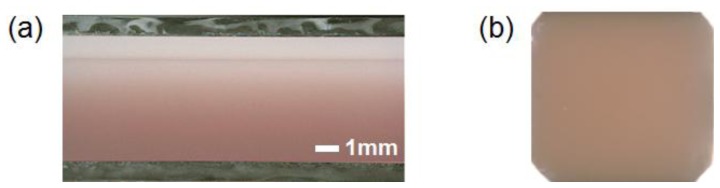
Photographs of tissue-like agar gel phantom for (**a**) cross-sectional view and (**b**) top view.

Before the *in vivo* experiments, preliminary experiments were carried out with tissue-like agar gel phantoms. Figure 3 shows the cross sectional photograph of the phantom. The phantom consisted of an epidermis and a dermis layer. An agar solution was prepared by diluting agar powder with saline. In order to make a base material with a scattering condition, Intralipid solution was added to the agar solution. A coffee solution was added into the base material as a substitute for melanin and this mixture was used to simulate an epidermis. Thus, the variable *C_c_* was used for the epidermis layer in the phantom experiments. Several materials, such as an India ink, have been used to mimic the absorption properties of melanin [29]. We first tried to mimic the absorption spectrum using an India ink solution. We investigated the absorbance spectrum of India ink (R591217, Rotring, Hamburg, Germany) based on the transmittance measurement in the preliminary experiment. The absorption spectrum of the India ink solution decays linearly as the wavelength increases, whereas that of melanin decays exponentially as the wavelength increases [30].

Therefore, it was difficult to reproduce the diffuse reflectance spectra of human skin over the visible wavelength range using India ink. Thus, we used a coffee solution to mimic the absorption spectrum of melanin. The coffee solution contained a brown pigment called melanoidin [31]. The absorption spectrum of melanoidin [32,33] has been reported to be similar to that of melanin. We observed the absorbance spectrum of coffee solution within a cuvette based on transmittance measurements and confirmed that the absorption spectrum of the coffee solution decays exponentially as the wavelength increases [30]. An oxygenated dermis was simulated by adding a small amount of fully oxygenated horse blood with a 44% hematocrit to the base material. Deoxygenated dermis was prepared by dropping a sufficient amount of Na_2_S_2_O_4_ saline solution on the surface of the oxygenated dermis. All of these layers were hardened in various molds having the required thickness and size by cooling at approximately 5.5 °C for 30 min. The thicknesses of the epidermis and dermis layer were 1.0 and 5.0 mm, respectively, while the area of each layer was 26 × 23 mm^2^. Details of preparing the phantoms and their optical parameters were published previously [8,30].

To confirm the validity of the proposed imaging method for visualization of the skin chromophore contents, *in vivo* experiments in the human skin were performed. A pressure cuff was applied to the upper arm of 5 subjects (all men, four Japanese and one Indonesian, mean age 27 ± 4 years). A cuff inflator was used to occlude the upper arm of the subjects as shown in Figure 1. After a rest of 300 s, acquisitions of RGB images and reflectance spectra were started and made for a total of 640 s at 5 s intervals. After 40 s of control measurements, the upper arm congesting cuff was inflated to 250 mmHg pressures for 300 s by a cuff inflator and then it was deflated for 300 s. Inflation of the cuff to 250 mmHg prevents blood flow from leaving the measurement site and also hinders arterial inflow. Analysis of both RGB images and reflectance spectra were made offline after measurements were completed.

## 4. Results and Discussion

**Figure 4.**
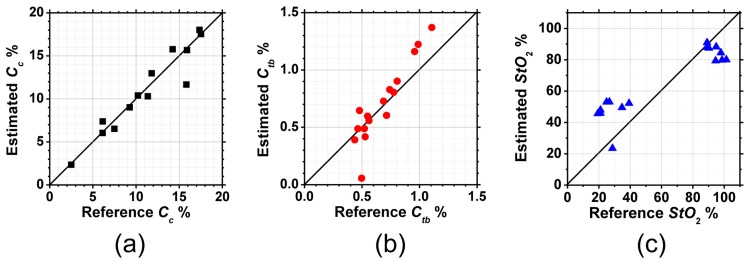
Comparison of the estimated values by the Wiener estimation and the reference values by spectrometer for (**a**) *C_c_*_,_ (**b**) *C_tb_*_,_ and (**c**) *StO*_2_ in the phantom experiments.

Figure 4 compare the estimated and reference values of *C_c_*_,_*C_tb_* and *StO*_2_. Average relative errors of *C_c_*_,_*C_tb_* and *StO*_2_ were 9.1, 17.5, and 54.5%, respectively. The estimated values of *StO*_2_ above 80% agree well with reference values but large discrepancy below 40% increases the average relative error.

**Figure 5.**
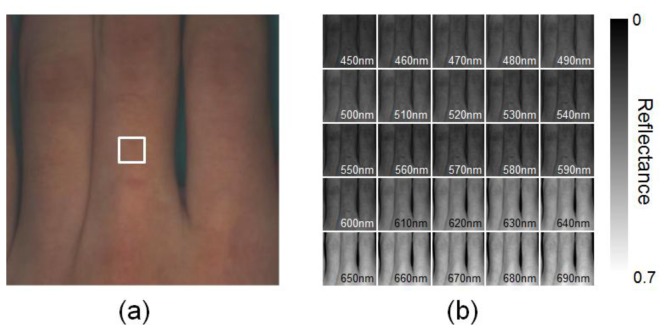
*In vivo* results obtained from human fingers under the normal condition for (**a**) raw RGB image and (**b**) the estimated images of spectral diffuse reflectance by the Wiener estimation.

**Figure 6.**
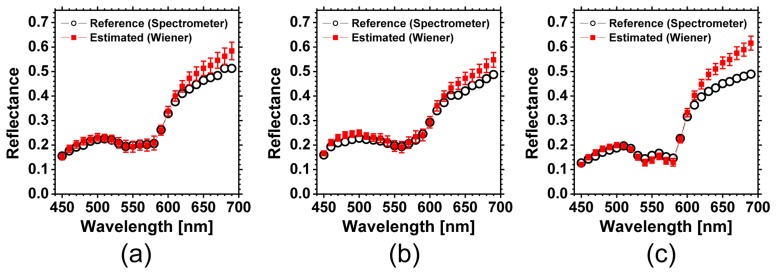
Comparisons of the estimated reflectance spectra averaged over the ROI (white square area in Figure 4) and the reference spectra measured by spectrometer for (**a**) pre-occlusion, (**b**) 300 s after occlusion, and (**c**) 90 s after deflation of pressure cuff.

Figure 5 shows an example of spectral images obtained from finger area of human hand in the normal condition. Spectral images of fingers were successfully obtained by WEM. Average values of the diffuse reflectance spectra over the region of interest, or ROI (white square area), are comparable to the measured spectra by the spectrometer, as shown in Figure 6. Spectral characteristics of hemoglobin for the different oxygenation state are well estimated. The values of GFC obtained from 5 subjects summarized in Table 1 indicate the accurate spectral reconstruction by WEM.

**Figure 7.**
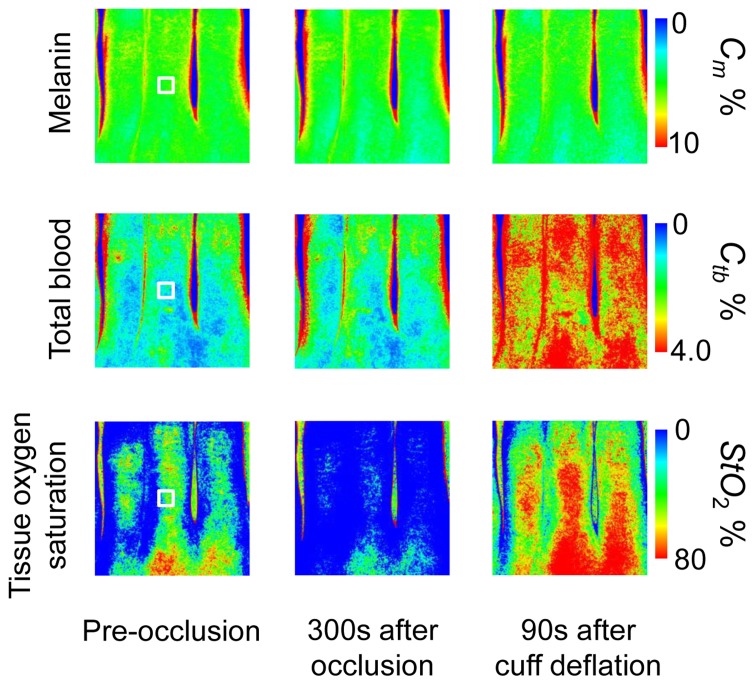
Typical images of fingers (from top to bottom; *C_m_*_,_*C_tb_*_,_ and *StO*_2_) for pre-occlusion, 300 s after occlusion, and 90 s after deflation of pressure cuff.

**Figure 8.**
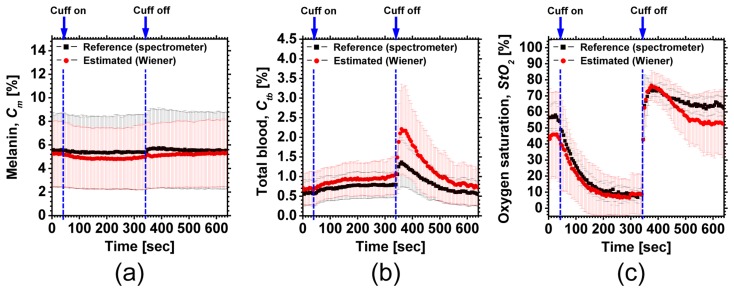
Time courses of (**a**) *C_m_*_,_ (**b**) *C_tb_*_,_ and (**c**) *StO*_2_ averaged over the ROIs (white squares in Figure 6) are compared with the reference values from the spectrometer recordings. The error bars show the standard deviations (*n* = 5).

Figure 7 shows an example of *in vivo* results during upper-arm occlusion. Time courses of *C_m_*_,_*C_tb_* and *StO*_2_ averaged over the ROIs (white squares are in Figure 7) for three subjects are shown in Figure 8. The average values of pre-occlusion *C_m_*_,_*C_tb_* and *StO*_2_ were 4.6 ± 3.7%, 0.8 ± 0.4%, and 44 ± 14%, respectively, which are close to typical normal values reported in the literature [8,14]. The average value of 44±14% for *StO*_2_ in pre-occlusion is lower than typical arterial oxygen saturation because the value of *StO*_2_ measured by this method represents oxygen saturation for the mixture of arterio-venous blood. After 300 s occlusion, *C_tb_* increased slightly and *StO*_2_ decreased remarkably, due to blockage of both venous and arterial blood flow. The value of *StO*_2_ exhibited the well-known deoxygenation curve, in which the oxygen saturation falls exponentially. The slight increase in *C_tb_* probably has a physiological cause. This is because, during occlusion, the venous outflow is reduced more than the arterial inflow. Immediately after the cuff was deflated, both *C_tb_* and *StO*_2_ increased sharply. These increases are assumed to have been due to post occlusion reactive hyperemia. In spite of the remarkable changes in *C_tb_* and *StO*_2_, *C_m_* remained almost unchanged during the measurements. This tendency of the hemodynamic response is also demonstrated in the results obtained from the measured spectra by the spectrometer.

Deviations of *C_tb_* from the reference values after cuff deflation are due to underestimations of reflectance at 540, 560, and 580 nm by WEM as shown in Figure 7. Considering noise in the camera response for **W** or using higher order terms of elements in vector **v** may improve the estimations of reflectance spectra [24,25]. In this study, we used the known spectral profiles of illuminant and camera. When the matrix **F** is unknown and experimentally determined, this method will be sensitive to small errors of measurement for determining the matrix **F**. Those issues should be investigated in future work.

This method should integrate all information along the depth direction because it relies on the steady-state diffuse reflectance. Therefore, the imaging system does not resolve the depth of the measurement. D’Alessandro *et al.* [34] have developed the technique separating melanin from oxygenated hemoglobin and deoxygenated hemoglobin through multispectral imaging combined with a voxel-based parallel processing Monte Carlo simulation under the condition of Nevoscope geometry and showed that subsurface oxygen saturation of localized region could be estimated with good implications for the reconstruction of three dimensional skin lesion volume. Such an approach based on the dependence of diffuse reflectance on the distance between the skin surface and localized chromophores will be useful for extending the method to depth-resolved imaging technique.

We used a single thickness for the epidermis and the dermis and only one typical wavelength-dependent scattering value and anisotropy for skin in the Monte Carlo simulation to derive the regression Equations (14) and (15) for the concentrations of melanin *C_m_* and total blood *C_tb_*. We also assumed a single thickness for the epidermis and the dermis in the Monte Carlo simulation. Diffusely reflected light from the skin will increase as the absorption of light in skin becomes small and will increase as the scattering coefficient *μ_s_* (*λ*) becomes small. Thus, if the scattering coefficients *μ_s_* (*λ*) of a sample are larger than the typical values used in the Monte Carlo simulation, the regression model will underestimate the chromophore concentrations. Diffusely reflected light from the skin will also increase as the anisotropy factors *g*(*λ*) decrease. Therefore, if the anisotropy factors *g*(*λ*) of a sample are larger than the typical values, the regression model will underestimate the chromophore concentrations. The probability that light is absorbed by melanin in the epidermis will be higher than the probability that light is absorbed by blood in the dermis as the epidermis becomes thicker. Therefore, if the thickness *t* of the epidermis is larger than the typical values, then the concentration of melanin in the epidermis or that of blood may be under- or overestimated, respectively. Hence, the measurements could be varied with the changes in the scattering coefficients *μ_s_* (*λ*), the anisotropy factors *g*(*λ*), and the thickness *t* of each layer. Studies on the Monte Carlo simulation model with the variations in the scattering coefficients, the anisotropy factors, and the thickness of each layer would strength the method in the future. We will investigate the regression equations involving those variations further in future work. Although there were some artifacts due to the shade originating from the curved and irregular surface of fingers, lateral distributions of *C_m_*_,_*C_tb_* and *StO*_2_ and their responses to the occlusions were successfully visualized.

## 5. Conclusions

In summary, a method for estimating melanin concentration, blood concentration, and tissue oxygen saturation in human skin based on spectral reflectance images reconstructed from a single snap shot of an RGB image by WEM is demonstrated in the present report. Experiments with phantoms demonstrated the validity of the proposed imaging method for visualization of chromophore contents. *In vivo* experiments confirmed the ability of the method to monitor chromophore contents and physiological changes in human skin tissue. The results of the present study demonstrate the possibility of evaluating the hemodynamics of subsurface skin tissue. The great advantages of this method are its simplicity and applicability, because the only devices required are a digital RGB camera with a known color profile, a white light source, and a personal computer. The total cost of our system was approximately $4,000. We expect to further extend this method in order to investigate the physiological and clinical indices of blood flow, such as the arterial inflow rate, the venous capacitance, and the maximum venous outflow.

## Figures and Tables

**Table 1.** The goodness-of-fit coefficient (GFC) obtained from the 5 subjects.

**Goodness-of-Fit Coefficient (GFC)**						
Subject	Mean	±SD	Max	Min	Number of spectra	Accuracy
#1	09993	0.0005	0.9999	0.9983	133	Good
#2	0.9980	0.0017	0.9998	0.9930	147	Colorimetrically accurate
#3	0.9993	0.0006	0.9999	0.9970	124	Good
#4	0.9993	0.0007	0.9999	0.9965	124	Good
#5	0.9997	0.0004	0.9999	0.9986	124	Good
